# Phylogenomic Placement of American Southwest-Associated Clinical and Veterinary Isolates Expands Evidence for Distinct *Cryptococcus gattii* VGVI

**DOI:** 10.3390/microorganisms10081681

**Published:** 2022-08-20

**Authors:** Juan Monroy-Nieto, Jolene R. Bowers, Parker Montfort, Guillermo Adame, Constanza Giselle Taverna, Hayley Yaglom, Jane E. Sykes, Shane Brady, A. Brian Mochon, Wieland Meyer, Kenneth Komatsu, David M. Engelthaler

**Affiliations:** 1 Pathogen and Microbiome Division, Translational Genomics Research Institute, Flagstaff, AZ 86005, USA; 2 Arizona Department of Health Services, Phoenix, AZ 85007, USA; 3 Instituto Nacional de Enfermedades Infecciosas “Dr. Carlos G. Malbrán”, Buenos Aires C1282AFF, Argentina; 4 Department of Medicine & Epidemiology, School of Veterinary Medicine, University of California-Davis, Davis, CA 95616, USA; 5 Department of Pathology, College of Medicine–Phoenix, University of Arizona, Phoenix, AZ 85721, USA; 6 Infectious Diseases Division, Banner Health/Sonora Quest Laboratories, Phoenix, AZ 85006, USA; 7 Sydney Medical School, University of Sydney, Sydney, NSW 2050, Australia; 8 Curtin Medical School, Curtin University, Perth, WA 6102, Australia

**Keywords:** *Cryptococcus*, whole-genome sequencing, VGVI, phylogenomics, molecular type

## Abstract

Whole-genome sequencing has advanced our understanding of the population structure of the pathogenic species complex *Cryptococcus* *gattii*, which has allowed for the phylogenomic specification of previously described major molecular type groupings and novel lineages. Recently, isolates collected in Mexico in the 1960s were determined to be genetically distant from other known molecular types and were classified as VGVI. We sequenced four clinical isolates and one veterinary isolate collected in the southwestern United States and Argentina from 2012 to 2021. Phylogenomic analysis groups these genomes with those of the Mexican VGVI isolates, expanding VGVI into a clade and establishing this molecular type as a clinically important population. These findings also potentially expand the known *Cryptococcus* ecological range with a previously unrecognized endemic area.

## 1. Introduction

Whole-genome sequencing has provided an important element in defining the population structure for many human fungal pathogens, including the *Cryptococcus* species complexes (*C. gattii* and *C. neoformans*). However, genomic tracking of *Cryptococcus* is seldom done for the estimated 223,100 annual clinical meningitis cases worldwide [1]. This dearth is even more pronounced for veterinary cases. Epidemiological and academic research efforts, using methods such as restriction fragment length polymorphism (RFLP), multilocus sequence typing (MLST), and, most recently, whole-genome sequencing [2], have defined the *Cryptococcus* subpopulations into molecular types and proposed them as separate species [3]. The most prevalent *C. gattii* populations that cause illness have been characterized and sequenced, and comprise mostly the molecular types VGI, and VGII, although the VGIII [4] and VGIV [5] genomic populations have also been well described. Because of increased collection and genome sequencing of isolates around the world, emerging and less prevalent populations have recently been added to the species phylogenomic tree. Such is the case for *C. gattii* molecular type VGV, which was found via environmental sampling in central Zambian woodlands [6], as well as the new lineage VGVI, which is represented by multiple laboratory strains derived from one or two clinical cases in Mexico from the early 1960s [6] and an isolate from a Mexican immigrant who lived in Spain from 1987 [7]. These clonal VGVI isolates were previously proposed as a distinct species, named *C. decagattii* [3].

We sequenced four clinical isolates and one veterinary isolate of *C. gattii* collected from 2012 to 2021 in the southwestern United States and Argentina. Here, we present the genomes of these isolates and their phylogenomic placement in the species tree.

## 2. Materials and Methods

Of the five isolates of *C. gattii* that were sequenced in this study, three were collected from patients with pulmonary or meningeal cryptococcosis in Arizona and classified as *C. gattii* by matrix-assisted laser desorption/ionization-time of flight (MALDI-TOF) mass spectrometry (MS) (Table 1). The geography and coincidence of the clinical cases prompted further investigation. The remaining two isolates were selected for sequencing from the Westmead Medical Mycology Collection, Sydney/Perth, Australia, due to their MLST profiles. These isolates were collected from a clinical case in Argentina [8] and a veterinary case in Arizona (Table 1).

Genome libraries were prepared with a DNA prep kit (Illumina) at one-fourth of the reaction volumes and sequenced on a NextSeq 550 (Illumina). Molecular typing was performed over the sequence reads via a custom 31-mer Kraken2 database [10] consisting of publicly available genomes from representatives of each molecular type of the *C. gattii* species complex, including the “VGVI” genomes that originated from Mexico in the 1960s and 1987 (SRA: SRS1519517, SRR3707827, SRR7345539). Read files for each sample were considered typed when read counts assigned to the top match in the custom database surpassed those of the second-best match by a factor greater than two. Draft genomes were assembled for use as references for single nucleotide polymorphism (SNP) detection, using SPAdes v3.10.1 with the “careful” flag activated [11].

Subsequently, sequence reads were compared to public genomes, including that of their putative molecular type VGVI (AZ04665 draft assembly used as alignment reference) by SNP-based phylogenetic inference. Reads were mapped to reference genomes using BWA v0.7.17 [12] and SNPs called using GATK UnifiedGenotyper v3.7 [13] using the bioinformatic pipeline NASP [14]. The SNP matrix output was filtered to include only positions where all genomes had ≥10X depth of reads and a read agreement proportion of ≥0.9 to produce high-confidence SNPs. The concatenated SNP profiles of each genome were used to perform maximum parsimony inference using Phangorn [15] to generate phylogenomic trees, using the ratchet method [16] from a 75% consensus of 30 ratchet trees that were produced from initial topologies found via random generation or random addition. Five hundred bootstrap replicates generated the confidence values for each node. Final phylogenies were plotted using ggtree [17]. Sequence data from this project are available in NCBI Bioproject PRJNA826887.

## 3. Results

**Figure 1:**
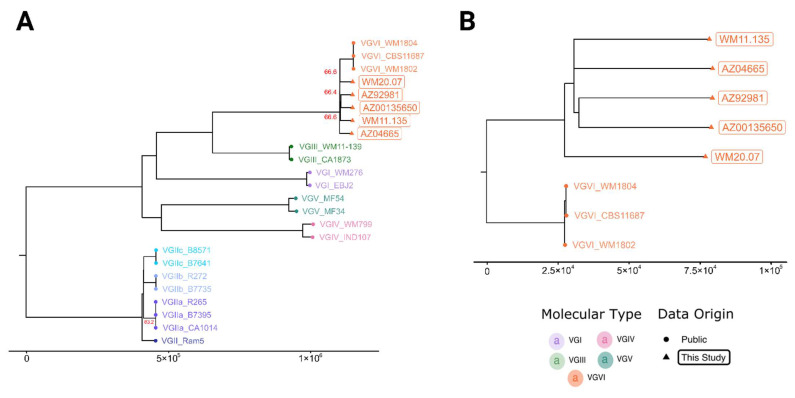
Maximum parsimony phylogenies based on high confidence SNPs showing boxes around the isolates sequenced in this study. (**A**) This analysis of 24 genomes includes two to eight publicly available genomes from each of the major *C. gattii* molecular types. The tree covers 87.40% of the reference and comprises 1,733,389 polymorphic loci, with a consistency index of 0.88 and a retention index of 0.97. (**B**) The VGVI-only phylogeny includes all eight available genomes for this molecular type and comprises 1,123,371 polymorphic loci covering 91.88% of the reference genome. This phylogeny has a consistency index of 0.78 and a retention index of 0.73. The reference for both trees was AZ04665 draft assembly (17.5 Mbp N50: 185.1 Kbp). All branch bootstrap values equal 100, except where noted in red numerals.

Genomes for the five isolates matched the VGVI reference genomes via k-mer content classification. Phylogenomic reconstruction confirmed that they were more related to the originally named VGVI isolates [6] than other *C. gattii* molecular types (Figure 1A). Mean pairwise distances within the VGVI clade were ~11.5 times shorter than the mean pairwise distance to the tips of the next major molecular type (VGIII), establishing VGVI as reciprocally monophyletic and distinct from other populations. The phylogenomic analysis of the VGVI genomes revealed approximately one hundred thousand high-confidence SNPs among them (Figure 1B); the long branches indicated a population structure with a deep (i.e., not recent) evolutionary history. The Argentinian genome WM 20.07 was basal to the rest of the recently collected samples yet more related to these than to the older WM 1804, WM 1802, and CBS 11687 collected from Mexican patients.

## 4. Discussion

In recent years, outbreaks of fungal disease affecting humans and animals have manifested previously uncharacterized taxa and challenged our knowledge of the epidemiology and biogeography of fungal disease. Such was the case for the global emergence of *Candida auris* [18] and for the appearance of *C. gattii* in the Pacific Northwest [4,19]. The five isolates sequenced in this study, four of which originate from human/veterinary clinical cases in warm, arid regions of the southwestern desert in the United States, similarly expand *C. gattii* taxonomy and potentially its biogeography by establishing VGVI as a distinct population of *C. gattii* with a long evolutionary history.

The origins of the isolates comprising VGVI suggest a new endemic area that may include the southwestern United States and Mexico, as well as regions of Argentina. If indeed this population inhabits the American Southwest, several questions about adaptations of VGVI to warm, arid environments are raised, including whether VGVI has departed from traditional niches for *C. gattii*, which is usually found in moist and nutrient-rich microenvironments. Dry soils in temperature-extreme environments are generally unexpected places to find fungi [20]; however, microenvironments such as the extensive cryptobiotic grounds in the southwestern United States [21,22] and organism adaptations such as those of *Coccidioides* spp.—both at the cellular [23,24] and life cycle levels (e.g., the soil sterilization hypothesis [25,26] and the small-mammal reservoir hypothesis [27,28])—illustrate the clear possibility of the presence of other cryptic environmental fungi in these ecosystems. Additionally, other desert-adapted yeasts exemplify survival strategies that may be suited to *C. gattii*. Such is the case for the cactophylic *Sporopachyderma* spp., which have been collected in southern Arizona, dwelling in saguaro cactus soft rot pockets [29,30], and which have been reported to be rare opportunistic human pathogens [31,32]. Of note, all known VGVI isolates were collected exclusively from clinical or veterinary infections. Environmental isolation will be an important step in elucidating the niche of VGVI and its endemicity in the region.

The basal placement of the Argentinian isolate in the VGVI phylogeny shows the immediate relation of the isolate to the North American cases and indicates that *C. gattii* VGVI is a possible risk for patients in previously undescribed endemic regions of the globe. A lack of clear exposure history for the Arizona human patients prevented confirmation of endemic locales at this time. Nevertheless, the link to the southwestern United States was compelling when the provenance of the isolates was considered; for example, clinical isolate 7685027, collected in southern California, and reported by Springer et al. [33], likely belongs to this clade, as its MLST profile is very similar to that of CBS 11687 [3]. The locale of the veterinary cases is of extreme interest, due to limited travel distances for pets. An additional veterinary case of *C. gattii* in Arizona was previously published [7], which may lead to additional epidemiological follow-up. Further genomic analysis of potential VGVI isolates, especially veterinary and environmental isolates, may clarify the biogeography, epidemiology, and potential health risks of this molecular type of *C. gattii*.

The VGVI population structure showed substantial differences across samples (Figure 1B), which contrasted with the close relatedness of the original three VGVI genomes. According to metadata published in 2003 [7], the samples WM 1802 (LA 390, INDRE 5604) and WM 1804 (LA 392, INDRE 5606)) were collected in 1961 and 1965, respectively, from disparately aged patients (i.e., 38 and 40 years of age), whereas published records for CBS 11687 (IHEM 14941S) indicated collection in 1987 in Europe from a Mexican immigrant [3,9]. This contradicts a previous hypothesis that proposed that these samples were derived from a single isolate [6]. Additional high-resolution SNP analysis of only these three genomes (data not shown) resulted in 144 and 149 high-confidence SNPs between CBS 11687 and the other two isolates, which in turn are differentiated by 21 bases between them. These relatively few differences explain the reduced diversity measurements for VGVI and provide further evidence that isolates either represent a single originating clone with subsequent laboratory acquired mutations, or independently originated from the same exposure source. In contrast to the reduced genomic diversity of the isolates that first defined VGVI, the three clinical isolates recently collected in Arizona in 2019 and 2021 were divergent, with ~100,000 high-confidence SNPs between samples. This reinforced the case for a deep evolutionary history that we have yet to uncover and underlined the likelihood of past clinical cases that were mischaracterized. 

*Cryptococcus* molecular typing is rare in clinical settings, but new technologies now make it a possibility. The use of MALDI-TOF MS in the Arizona clinical microbiology laboratory in this study is a recent change. Previous methods may have misclassified *C. gattii* infections as *C. neoformans*, which is the more likely species to be encountered in this setting. The use of MALDI-TOF MS for rapid, simple, and reliable molecular type identification in *Cryptococcus* spp. was demonstrated in research applications [3,34,35]; MALDI-TOF MS could potentially be used to rapidly screen for suspect VGVI isolates. Better isolate typing is an important development that may spark further research, providing concurrent and retrospective isolates to fill the gaps in our understanding of molecular type VGVI.

Tracking the geographical origin and the dynamics of spread is especially challenging in fungi, due to their complex ecology and the large timescales, usually millennia, that encompass the evolution of these organisms [4,36]. Reconstructing likely scenarios for spread is essential to the understanding of disease emergence and risks to public health; both natural history and human commercial activities have been correlated with the emergence of fungal etiologic agents and fungal resistance, such as in *Candida auris* [37,38] and *C. gattii* VGII [39]. These advances/theories are particularly reliant on whole-genome sequencing and molecular clock analyses that reveal the time scale and patterns of evolution; these efforts are ongoing for *C. gattii* VGVI. Further surveillance that includes typing methods, especially whole-genome sequencing, will provide the opportunity to clarify the endemicity of VGVI and to ascertain whether this pathogen represents an emerging risk to susceptible human and animal hosts in the identified regions.

## Figures and Tables

**Table 1:** Case information for *C. gattii* VGVI genomes. Definitions: CHF, congestive heart failure; CSF, cerebrospinal fluid; ND, not determined.

ID	Isolate Source	Collection Locale	Collection Year	Sample Type	Comorbidity	Cryptococcosis Presentation	WGS Source	Metadata Source
AZ04665	Clinical	USA/AZ	2019	CSF	HIV/AIDS	Meningitis	This Study	This study
AZ92981	Clinical	USA/AZ	2019	Blood	Alcoholic cirrhosis and hepatitis, CHF	Pulmonary	This study	This study
AZ00135650	Clinical	USA/AZ	2021	CSF	Pulmonary lesions/ Hx of Lymphoma	Bronchitis/ Meningitis	This study	This study
WM 11.135	Veterinary	USA/AZ	2011	Nasal	Possible underlying hepatopathy	Upper respiratory signs	This study	This study
WM 20.07	Clinical	Argentina/ Salta	2017	CSF	Malnutrition	Meningo- encephalitis	This study	[8]
WM 18.02	Clinical	Mexico/DF	1961	CSF	ND	Meningitis	[4]	[7]
WM 18.04	Clinical	Mexico/DF	1965	CSF	ND	Meningitis	[4]	[7]
CBS 11687	Clinical	Mexico *	1987	ND	ND	ND	[6]	[9]

* The isolate was obtained from a Mexican non-HIV patient living in Spain.

## Data Availability

All sequence data generated and used are made public through NCBI. Sequence reads produced for this study can be found in Bioproject PRJNA826887.
